# Transplanting Large Portions of Skin on Fresh Wounds

**Published:** 1885-08

**Authors:** 


### Selections.

### Transplanting Large Portions of Skin on Fresh Wounds, by Esmarch.

Translated from the German by Floyd S. Crego, M. D.

Lecturer on Diseases of Nervous System and Insanity, Medical Department, Niagara University.

I call your attention to the transplanting of large portions of skin on fresh wounds, which, within a short time, I have accomplished with great success, and especially in those cases where there was such a large loss of substance that formerly we were accustomed to leave them to heal by granulation. To this class belong especially the large wounds which are left on the forehead after rhinoplasty, and on the face in other plastic operations and large wounds, which occur on the scalp from accident by machinery, etc.

This method of transplanting skin (dermana plastie) is, as you know, not new. As long ago as 1823, Burger, in Marburg, grafted a large portion of skin on the nose, which had been removed from the thigh, but on account of the failure of other surgeons (Dzondi v. Graefe, Duffenbach v. Langenbeck), this method had no correct introduction till very recent times, when Wolff, of Glasgow, reported several cases, in which he had cured ectropion by grafting on the eyelid a piece of skin removed from the arm.

The treatment has been followed with good results by oculists (Zehender and others), and about six years since I obtained very good results in the cure of this trouble by this same operation. But it is only within a very few years that I began to make use of this treatment in a general way, and I am so pleasd with the result that I should like to recommend it to be tried in other large defects of the skin. The rapid healing by this operation of such defects is manifest. The first case I report is that of a woman who for ten years has suffered with cancer rodens on the bridge of the nose, and has twice before been operated on by scraping, followed by thermo-cauterization. The cancer had grown again during last summer, and was now as large as a ten pfennig piece (about as large as a five-cent piece). The borders were indurated, but the base of the cancer was still somewhat movable. On account of the double failure, the woman was in a very doubtful mood, and was afraid, and with cause, that the whole nose would be destroyed. In order to avoid the disfigurement which follows the removal of skin and tissue from the forehead, for the purpose of covering the nose, I proceeded as follows: cutting around the diseased portion quite into healthy tissue, dissecting it from the cartilage, I covered it at once with a portion of skin, the shape of a myrtle leaf, and about 3 cm. long and 1.50 cm. wide, which had been removed from the left arm. The wound of the arm was carefully closed with catgut ligature, and healed by first intention. The portion of skin which had been removed immediately contracted, by placing it on a flat surface, and I removed all the fat and cellular tissue very carefully with concave shears, and sewed the portion of skin to the four sides of the wound with a few fine catgut ligatures, so that it completely covered the wound without stretching. Over this was placed a piece of lint wet in sublimate solution. When this was removed, after ten days, the portion of skin was completely united and looked only somewhat paler and yellower than the rest of the strongly reddened skin of the nose. In the next months, there formed a layer of hardened epidermis, which it was necessary to soak off with warm water. In the meantime, the patch assumed a better color, and in four months did not look much differently than the skin surrounding it. In the words of the patient, "there was a decided change for the better." The second case is that of a pigmented nævus of the cheek, which was very ugly in appearance. They are often found, as in this case, near the outer angle of the eye, and disfigure the face so much. Formerly, in such cases, after the removal of the whole nævus, I have repaired the loss of substance by a flap, removed from the neighboring skin of the forehead or cheek. In one case, I allowed the wound to granulate, and then, to hasten the process, transplanted many small points of skin, after the method by Reverdin. But in this case, the result is much more comely. In this case, after the removal of the nævus, I covered the wound at once with two portions of skin, the shape of a lance, which had been removed from each arm, and with the catgut ligature sewed them to the free borders of the wound. Here also, as before, all the wounds healed by first intent. The third case is of the same character, and the picture shows the face after fourteen days; as you see, I did not remove the pigmented skin from the eye-lid, but will remove that by a second operation in order not to complicate the case.

The fourth was that of a patient who was run over by a wagon, the wheel passing over the face. By this accident, the parts were very badly crushed. The upper jaw was broken and pressed out of shape. The nose was partially destroyed, and the skin covering it, and that adjacent to it, was peeled off. The thing to do was to close this hole, through which one could see into the recesses of the nose. For this purpose, I took the large flap from the forehead, and used it as indicated to cover this, and to build the nose. The wound of the forehead which was made by removal of the flap, was covered with skin removed from each arm; all these healed by first intent, and, as you see, a good result obtained.

Photographs and drawings of the cases were shown when the paper was read before the fourteenth Congress of the Society of German Surgeons.

For the success of this method, it is of the greatest importance that all the fat and cellular tissue be removed with the greatest care from the inner side of the portion of skin, after the method so accurately described by Wolff. The fat removed for transplanting can be placed on a flat surface, or it can be placed on the point of the left forefinger, and with the concave scissors cut away, with a number of fine cuts, all the tissue which is soft and has a yellowish appearance, until all the inner surface is quite smooth, and appears as white as a white kid glove. The flap should then be washed off once with solution of sublimate, and then placed carefully on the wound on which it is to be transplanted. To sew it very carefully to the edges is unnecessary, and I should not recommend it. It is enough to put in a few catgut ligatures to make sure it does not shrink. The flap must be made considerably larger than the surface of the wound to be covered, because the skin will, as you all know, become much smaller by shrinking. I would try to cover fresh scalp wounds, where there is an extensive loss of substance, in this manner. It is not necessary, of course, to remove the compensating flap from the patient himself. I have used for this purpose, before this, portions of skin removed from amputated limbs, and in one case I made use of the portion of skin which covered an enormous femoral hernia, which I had to cut away in an operation for radical cure.

